# Tracking NF-kB activity across steady-state neutrophil maturation

**DOI:** 10.1038/s41420-025-02737-w

**Published:** 2025-10-06

**Authors:** Nathan E. Jeffries, Daniel J. Floyd, Shenglin Mei, David B. Sykes, Michael K. Mansour

**Affiliations:** 1 Center for Regenerative Medicine, Massachusetts General Hospital, Boston, MA USA; 2 Division of Infectious Diseases, Massachusetts General Hospital, Boston, MA USA; 3 Fralin Biomedical Research Institute, Virginia Tech FBRI Cancer Research Center, Washington, DC USA; 4 Department of Biomedical Sciences and Pathobiology, College of Veterinary Medicine, Virginia Tech, Blacksburg, VA USA

**Keywords:** Cell death and immune response, Gene regulation in immune cells, Immune cell death, Neutrophils, Myelopoiesis

The transcription factor complex Nuclear Factor Kappa B (NF-kB) performs many important roles in hematopoiesis and immunology [1, 2]. In cells of the innate immune system, NF-kB acts downstream of pattern recognition receptors to regulate the expression of pro-inflammatory genes. The steady-state production of peripheral blood neutrophils depends on signaling through toll-like receptor 4, an upstream activator of NF-kB [3]. In mature neutrophils, NF-kB activation can increase survival following inflammatory stimuli [2]. While the importance of NF-kB for neutrophils in inflammatory conditions appears well-understood, the role of NF-kB in neutropoiesis and maturation at steady-state remains to be thoroughly elucidated. The inherent short lifespan of neutrophils complicates temporal analyses, and protein-based approaches to measuring NF-kB activation, which are destructive and rely on bulk cell lysates, do not account for heterogeneity in samples or permit isolation of cells based on their level of NF-kB activation.

To overcome these limitations, we created a new model cell line that enables non-destructive measurement of NF-kB activation during in vitro neutrophil maturation at single-cell resolution. A wild-type murine granulocyte-macrophage progenitor (GMP) line was established by stable expression of the estrogen-receptor HOXB8 (ERHOXB8) fusion protein as previously described [4]. In this system, GMP-like cells self-renew continuously in the presence of β-estradiol (E2) and differentiate synchronously into phenotypically mature, functional neutrophils over 3–5 days of culture in the absence of E2 [5]. Viral transduction was used to introduce a green fluorescent protein (GFP) reporter for NF-kB activity, consisting of GFP encoded downstream of an NF-kB binding site and minimal promoter [6].

From the transduced sample, GFP^LOW^ cells were sorted and returned to culture (Fig. 1A). A second round of sorting for cells GFP^POS^ after stimulation with PMA/ionomycin was used to collect cells that were capable of robust GFP induction. Clonal cell lines were generated by limiting dilution, and one NF-kB-inducible GFP (NG) clone was selected based on its low background and high sensitivity, as evaluated by overnight stimulation with lipopolysaccharide (Fig. S1A, B).

**Fig. 1:**
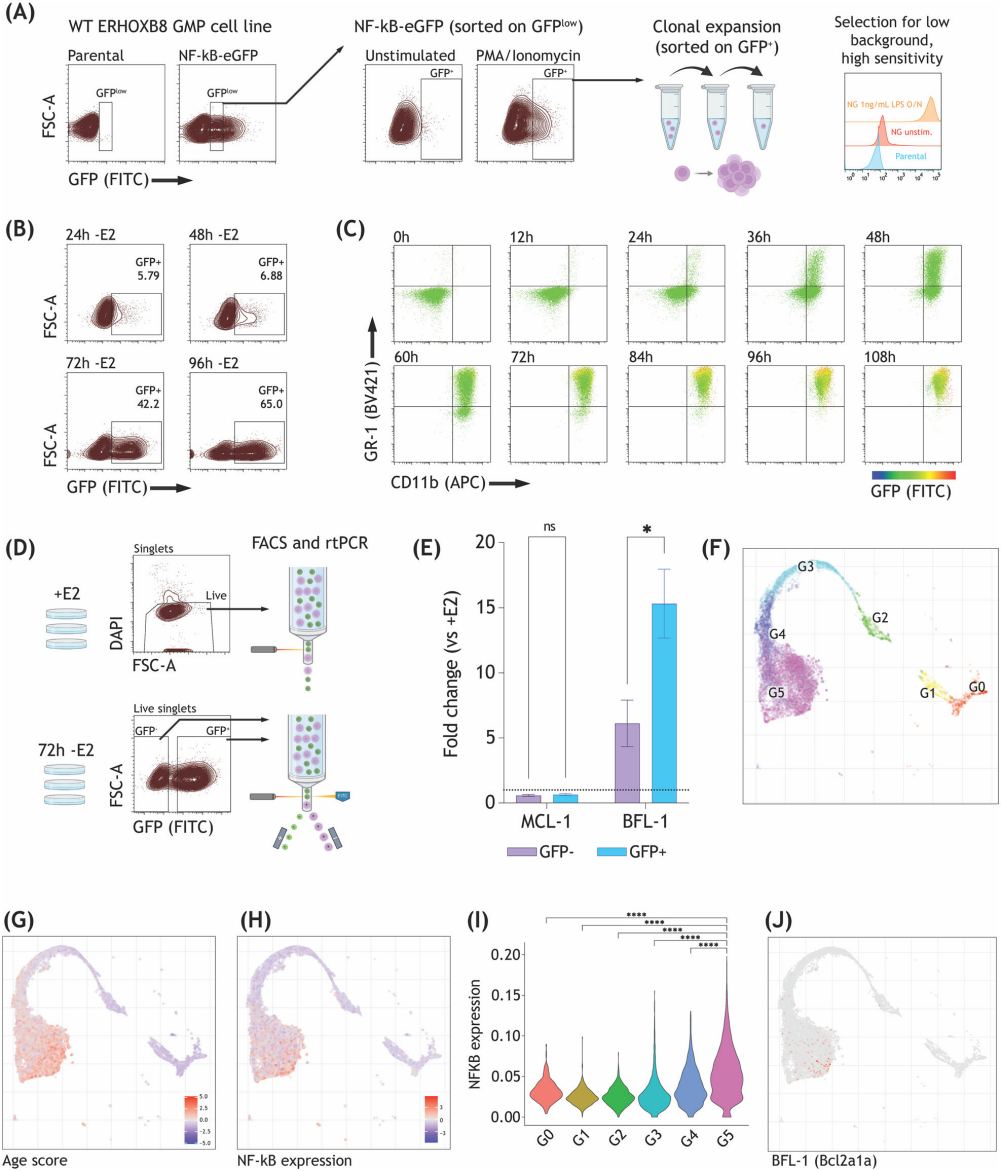
Mature neutrophils increase NF-kB activity and BFL-1 expression. **A** Method for generating a low background, high sensitivity NF-kB-inducible GFP (NG) reporter cell line. WT ERHOXB8 cells were transduced with NF-kB-GFP reporter virus, sorted for GFP^LOW^, then stimulated with 100 nM PMA and 100 nM Ionomycin prior to sorting for GFP^POS^. Cells from this population were expanded clonally by limiting dilution and clones were evaluated for GFP expression following overnight stimulation with 1 ng/mL LPS. FACS plots show live singlets. **B** NG cells were removed from β-estradiol (E2) at 24 h increments and analyzed for GFP positivity. Shown are singlets. **C** Same experiment shown in (**B**), but at 12 h increments and multiplexed with surface expression of GR-1 and CD11b. GFP positivity is indicated using the heatmap axis. **D** Sort strategy for gene expression analysis. +E2 cells were sorted from live singlets; 72 h -E2 cells were sorted from live singlets and based on GFP positivity. **E** Expression of BCL-2 family members in GFP^POS^ and GFP^NEG^ -E2 cells normalized to +E2 expression. *N* = 3 independently cultured replicates of the same cell line, comparisons made using a paired, parametric t-test with multiple comparisons corrected for using the Holm-Šídák method (MCL-1: *p* = 0.093, BFL-1: *p* = 0.009), *p ≤ 0.05, ns (not significant) *p* > 0.05. Shown is mean ± SD. **F** UMAP embedding showing neutrophil differentiation and maturation trajectories from G0 (GMP) to G5 (mature neutrophil). Single cell data was obtained from GSE137540. Scaled average expression of neutrophil aging signatures (**G**) and NF-kB signatures (**H**) visualized on UMAP embedding. **I** Violin plots of the NF-kB gene signature score in different neutrophil subsets. Significance was assessed using two-sided Wilcoxon rank sum test (complete list of p-values in Table S2), ****p < 0.0001. **J** UMAP visualization of BFL-1 expression. **A**, **D** Graphics were created with BioRender.com.

A time course was used to assess NF-kB activation during the process of maturation from GMP to neutrophil. NG cells that were removed from E2 at 24 h increments and evaluated for GFP expression using flow cytometry demonstrated a shift in NF-kB activation starting after 48 h of maturation and continuing until 96 h (Fig. 1B). Figure 1C shows the same analysis at 12 h increments multiplexed with surface expression of CD11b and GR-1, both expressed in the mature neutrophil state only, with GFP positivity indicated by the heatmap. Here, it appears that the onset of NF-kB activation occurs after cells express both surface markers and are, therefore, phenotypically mature by flow cytometry. The proportion of positive cells peaked at 96 h and was maintained through 108 h (Fig. S2).

To address whether the onset of NF-kB activation in neutrophils was associated with a change in mature effector function, NG cells removed from E2 at 12 h increments were assessed for their ability to phagocytose iRFP-labeled *Candida albicans* (Fig. S3A). The ratio of cells capable of phagocytosis to cells that did not phagocytose *C. albicans* was similar for GFP^POS^ and GFP^NEG^ cells, suggesting that cells with increased constitutive NF-kB activation do not have an inherent functional advantage when challenged with this pathogen (Fig. S3B). Additionally, the total proportion of GFP^POS^ cells did not change upon the addition of *C. albicans*, though this is possibly explained by the short incubation time (30 minutes) (Fig. S3C). We also assessed the expression of the cell surface markers and regulators of neutrophil migration CXCR2 and CXCR4 in relation to NF-kB activation. For both CXCR2 and CXCR4, no apparent relationship to NF-kB was observed (i.e., the proportion of cells positive for each marker appeared similar for GFP^POS^ and GFP^NEG^ cells) (Fig. S4A, B).

Next, we sought to identify whether the change in NF-kB activation was associated with a change in the expression of pro-survival genes, as regulation of apoptosis is known to be a function of NF-kB in mature neutrophils [1, 7]. We measured the expression of two BCL-2 family members, both implicated in the regulation of survival in neutrophils, in NG neutrophils sorted by GFP positivity: MCL-1, a pro-survival gene regulated at the protein level to serve as a negative control, and BFL-1, a pro-survival gene regulated transcriptionally and upregulated in response to GM-CSF stimulation [8, 9]. Figure 1D shows the FACS sorting strategy used to isolate samples for RT-PCR. RNA expression in mature cells sorted based on GFP positivity was normalized to immature (+E2) cells sorted for viability. Strikingly, mature GFP^POS^ cells demonstrated approximately 2.5-fold greater expression of BFL-1 than mature GFP^NEG^ cells from the same culture (FC = 15.3 and 6.1, respectively) (Fig. 1E). As expected, MCL-1 expression did not differ significantly between GFP^POS^ and GFP^NEG^ cells.

To assess whether the observation of increasing NF-kB activation and BFL-1 expression in neutrophils is merely a feature of our model system, we analyzed public data containing 19,582 primary cell transcriptomes and consisting of GR-1^POS^ cells sorted from murine bone marrow (BM), peripheral blood, and spleen, and KIT^POS^ cells sorted from BM (Fig. 1F) [10]. Cells were grouped into 6 clusters, annotated G0-G5, with G0 corresponding to immature progenitor cells (expressing CD34 and KIT) in the BM, G1-G4 corresponding to maturing neutrophils present mostly in the BM, and G5 corresponding to mature peripheral blood and splenic neutrophils. Both age signature and NF-kB pathway gene expression appeared to increase for cells in the G5 mature neutrophil cluster as compared to the progenitor cell cluster, with G5 neutrophils demonstrating the highest levels of NF-kB pathway expression (Fig. 1G–I). BFL-1 expression, although scant, was generally limited to mature neutrophils (Fig. 1J).

BFL-1 is a direct transcriptional target of NF-kB and is expressed at high levels in human peripheral blood neutrophils [11, 12]. Here, we observed an increase in BFL-1 expression in mature ERHOXB8 neutrophils as compared to immature progenitors, with the highest expression seen in cells with increased constitutive NF-kB activity. Our study only assesses these parameters in one instance – wild-type ERHOXB8 neutrophils in steady-state conditions – and therefore does not imply a causative relationship between NF-kB activation and BFL-1 expression. Given this limitation, these findings do suggest a positive correlation between NF-kB activation and BFL-1 expression in neutrophils. Notably, analyses using human HL60 cells have observed a similar increase in BFL-1 expression during neutrophilic maturation, and genome-wide knockout of the primary murine isoform of BFL-1 can result in increased neutrophil apoptosis in inflammatory conditions [12, 13]. Our data suggest that BFL-1 expression in neutrophils is upregulated temporally in steady-state conditions, and underscore the importance of further investigation to determine whether NF-kB mediates this process.

Outside of our study, we hope the development of an ERHOXB8 GMP cell line with an NF-kB-inducible GFP reporter might serve as a valuable resource for other investigators. Our manuscript also highlights the utility of the ERHOXB8 system in the study of neutrophil maturation, demonstrating that it can recapitulate features of granulopoiesis in vitro. We acknowledge that our study has several limitations. The transcriptomic dataset analyzed here does not sufficiently support the correlation between NF-kB activation and BFL-1 expression observed in vitro; this should be assessed through additional in vivo experimentation. We also recognize that our experimental approach, which relies on the assessment of two distinct parameters in a single biological instance and does not include a null condition, only allows for the identification of a correlative relationship between NF-kB activity and BFL-1 expression; experimental validation, for example, through deletion of the NF-kB regulatory element upstream of BFL-1, is necessary to determine whether NF-kB is a direct regulator of BFL-1 expression in this context. Measurement of BFL-1 expression in response to treatment with chemical activators and inhibitors of NF-kB may also be useful. Ultimately, identifying the mechanism by which constitutive NF-kB activity is increased in neutrophils may contribute to our understanding of neutrophil lifespan and survival.

## Supplementary information

Supplementary fig. S1

Supplementary fig. S2

Supplementary fig. S3

Supplementary fig. S4

Supplement
