# Supplementary figures and images for: Tracking NF-kB activity across steady-state neutrophil maturation
Source: Cell Death Discov. 2025 Oct 6;11:437. doi: 10.1038/s41420-025-02737-w (PMC12500873; doi:10.1038/s41420-025-02737-w)

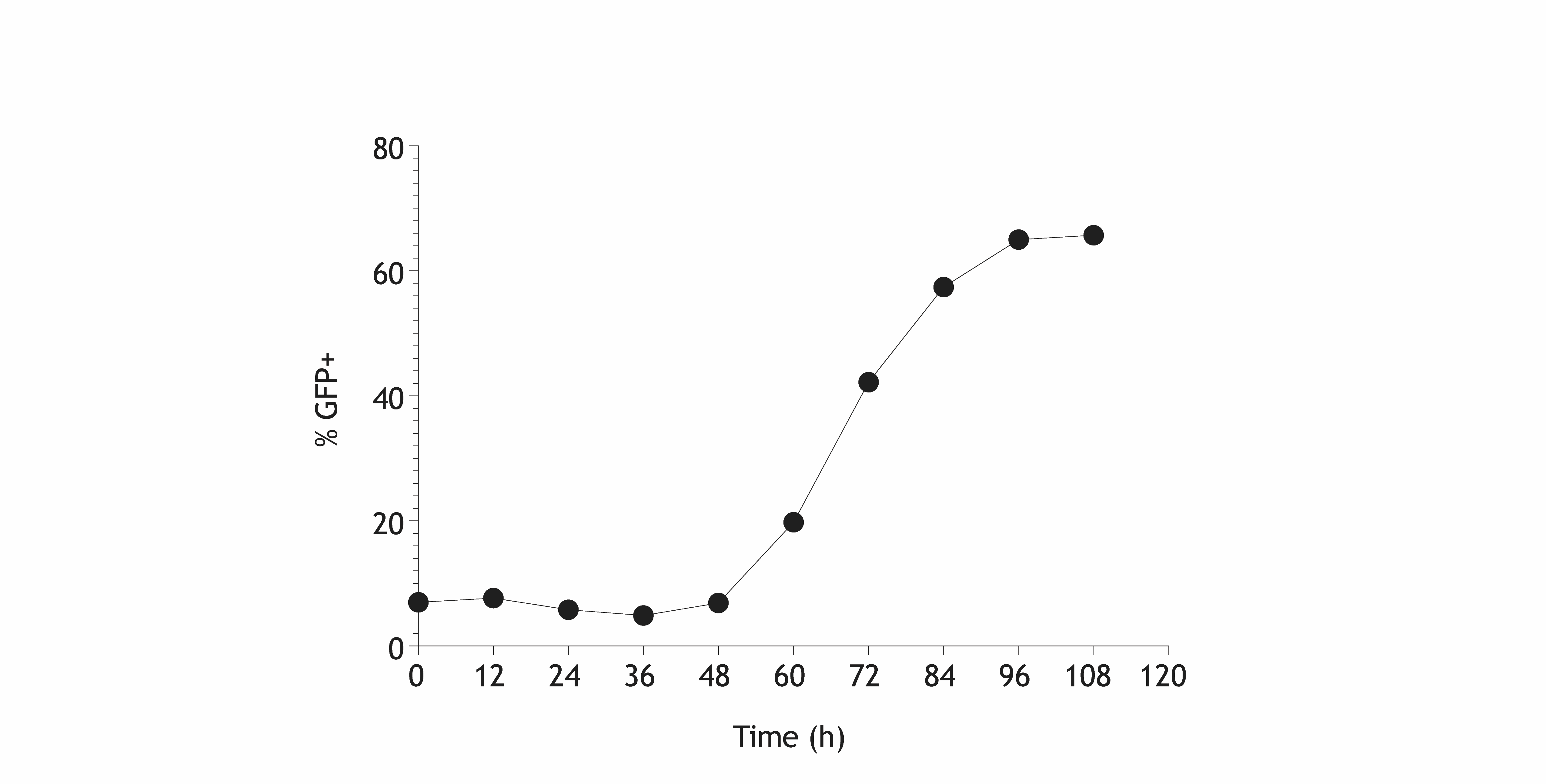

Supplement: Supplementary file 2 — Supplementary fig. S2 [file 41420_2025_2737_MOESM2_ESM.tif]
