# Supplementary material for: Tracking NF-kB activity across steady-state neutrophil maturation
Source: Cell Death Discov. 2025 Oct 6;11:437. doi: 10.1038/s41420-025-02737-w (PMC12500873; doi:10.1038/s41420-025-02737-w)
Supplement: Supplementary file 5 — Supplement [file 41420_2025_2737_MOESM5_ESM.docx]

**Title**

Tracking NF-kB activity across steady-state neutrophil maturation

**Authors**

Nathan E. Jeffries, Daniel J. Floyd, Shenglin Mei, David B. Sykes, Michael K. Mansour

1. **Supplemental materials and methods**
   1. Reagents

PMA was acquired from MedChemExpress (HY187395MG), dissolved to a stock concentration of 10 mM in dimethyl sulfoxide (DMSO) (Sigma D8418) and used at a final concentration of 100 nM. Ionomycin was acquired from Santa Cruz Biotechnology (sc-3592), dissolved to a stock concentration of 10mM in DMSO and used at a final concentration of 100 nM. Lipopolysaccharide (LPS) was dissolved to a stock concentration of 1 mg/mL in H2O and used at a final concentration of 1 ng/mL or 0.5 ng/mL as indicated. FACS buffer was composed of phosphate-buffered saline (PBS) (Corning 21-040-CV) with 2% fetal bovine serum (FBS) (Gibco A31605-01) and 1 mM EDTA (Invitrogen AM9260G). cRMPI for phagocytosis assays was composed of 1640 RPMI (Corning 10-104-CV) with 10% heat-inactivated FBS (Gibco A5256801) 100 U/mL PenStrep (Gibco 15-140-148) and 2mM L-Glutamine (Gibco 25030081).

- 1. Cell lines

Wild-type ERHOXB8 cell lines were generated from CD45.1 STEM mice as previously described^1,2^. The pSIRV-NF-kB-eGFP reporter plasmid was a gift from Peter Steinberger (Addgene plasmid #118093; http://n2t.net/addgene:118093; RRID: Addgene_118093)^3^. Virus was produced by transient transfection of 293T cells (ATCC CRL-3216) with the reporter plasmid and an equal mass of ecotropic packaging (EcoPack) plasmid using Lipofectamine 3000 (Invitrogen L3000-015). Transduction of ERHOXB8 cells was performed by 60-minute centrifugation at 1000g with 8ug/mL polybrene (VectorBuilder PL0001). Cells were maintained in RPMI with L-glutamine (Corning 10-040-CV) supplemented with 10% FBS, 1% murine stem cell factor (SCF) conditioned media, corresponding to a final SCF concentration of approximately 100 ng/mL, 100 units/mL Penicillin & 100 μg/mL Streptomycin (Gibco 15140-122), and 500 nM β-estradiol (Sigma E-2758, stock 10 mM dissolved in 100% ethanol). For differentiation assays, cells were cultured in the same media without β-estradiol. Cell lines were tested for mycoplasma at least every 6 months.

*C. albicans* SC5314 constitutively expressing far-red fluorescent protein (iRFP *C. albicans*) was kindly donated by Robert Wheeler (University of Maine, Orono, ME). *C. albicans*was grown in YPD liquid media (yeast extract, peptone, dextrose) containing 1% yeast extract (Acros Organics, Fair Lawn, NJ, USA), 2% peptone (BD Biosciences, San Jose, CA, USA), and 2% dextrose (Sigma-Aldrich). C. albicans was cultured overnight at 30 °C on a rotating culture wheel (Thermo Fisher Scientific). The following day, *C. albicans* was removed from the wheel, washed twice with PBS and resuspended in PBS. *C. albicans*was counted using the LUNA automatic cell counter (Logos Biosystems, South Korea) and kept on ice until the time of the assay.

- 1. Flow cytometry

Transduced cells were initially sorted for GFP positivity on a Bigfoot Cell Sorter (Thermo Fisher Scientific, Waltham, MA, USA) and for response to PMA/Ionomycin stimulation on a Becton-Dickinson FACSAria III (BD Biosciences, Franklin Lakes, NJ, USA). Cells assessed for response to LPS, GFP expression during differentiation, surface marker expression, and phagocytosis were analyzed on a BD FACSCelesta, and cells were sorted for gene expression analysis on a BD FACSAria I. A minimum of 10,000 singlets was collected for each sample analyzed; where indicated, DAPI was used to assess viability, and 10,000 live singlets were collected. For surface marker analysis, cells were stained for 30-minutes at 4°C in FACS buffer, washed once in FACS buffer, and resuspended in FACS buffer or FACS buffer with DAPI. A complete list of reagents used for flow cytometry can be found in **Table S1**.

- 1. Phagocytosis assay

Transduced cells were taken out of β-estradiol in 12-hour increments starting from 0 hours up until 108 hours for comparison of phagocytic functionality throughout maturation. Cells from each timepoint were resuspended at a concentration of 2e6 cells/mL in cRPMI. 50 uL pre-warmed cRPMI was added to each well of a 96-well plate and 50 uL of cells added to each well (100,000 cells per well). Resting wells received another 50 uL cRPMI while challenge wells received 50 uL cRPMI containing 400,000 yeast (multiplicity of infection = 4). Co-incubation of cells and *C. albicans* yeast proceeded for 30 minutes at 37°C and 5% CO_2_ before being placed on ice for 10 minutes to stop excess phagocytic activity. The plates were spun down at 550 x g for five minutes at 4°C and cell pellets were then stained with anti-mouse CD45.1 for 45 minutes in the dark at 4°C. Following staining, cells were washed and pelleted again before being resuspended in 150 uL cold FACS buffer. The plate was left on ice until just prior to data acquisition on a BD FACSCelesta with a blue, violet, red (BVR) laser configuration with specific wavelengths at 488 nm, 405 nm, 640 nm respectively. Total events were first gated by scatter to target cells of correct size and granularity of murine neutrophils. Forward and side scatter singlets were then gated before collecting 10,000 CD45.1^+^ events. Events positive within the AlexaFluor647 detector were deemed positive for phagocytosis.

- 1. Gene expression via real-time polymerase chain reaction (rtPCR)

RNA from sorted cells was isolated using the Qiagen RNeasy Micro Kit (74004) according to the manufacturer’s protocol. cDNA was generated using the Applied Biosystems High-Capacity cDNA Reverse Transcription Kit (Fisher Scientific 4368814) according to the manufacturer’s protocol. RT-PCR analysis was conducted using the Bio-Rad iTaq Universal SYBR Green Supermix (1725121) according to the manufacturer’s protocol. Primer sequences were originally identified using the Harvard PrimerBank (https://pga.mgh.harvard.edu/primerbank/) and are as follows: MCL-1 (forward: CAAAGATGGCGTAACAAACTGG, reverse: CCGTTTCGTCCTTACAAGAACA); BFL-1 (forward: GGCTGAGCACTACCTTCAGTA, reverse: TGGCGGTATCTATGGATTCCAC); β-actin (forward: GGCTGTATTCCCCTCCATCG, reverse: CCAGTTGGTAACAATGCCATGT).

- 1. Neutrophil single cell data analysis

Neutrophil single cell data and cell annotation were obtained from Xie et al^4^. We downloaded raw counts matrices of bone marrow single cell data from GSE137540, and low-quality cells with fewer than 600 total UMIs detected were filtered out. Next, we performed data normalization and dimensionality reduction using the Pagoda2 package^5^. We used conos to integrate multiple scRNA-seq datasets together (k=15, k.self=5, matching.method='mNN', metric='angular', space='PCA'). Principal component analysis was performed on 2000 genes with the most variable expression selected by conos^6^. A neighborhood graph was constructed, and the embedding was visualized using UMAP with default parameters. To characterize neutrophil maturation trajectory, G0–G5 stage annotations provided by Xie et al^4^ were projected onto the UMAP embedding (**Figure 1F**). This approach successfully reproduced the continuous trajectory of neutrophil maturation from early progenitors (G0) to mature neutrophils (G5), consistent with previous findings.

- 1. Gene set signature score in single cell data

We used a gene set signature score to evaluate cell states across various neutrophil subsets. These signature scores were determined by calculating the scaled average expression levels of genes within a specific set. Neutrophil aging signatures were sourced from Xie et al^4^. NF-kB pathway genes were derived from the GSEA database (BIOCARTA_NFKB_PATHWAY). Statistical significance was evaluated using the Wilcoxon rank-sum test, with p-values adjusted via the Benjamini-Hochberg (BH) method (**Table S2**).

- 1. Data analysis and statistics

Flow cytometry data were analyzed using FlowJo v10.10.0 (BD Biosciences, Ashland, OR, USA). RT-PCR data were analyzed using Bio-Rad CFX Maestro (Hercules, CA, USA). Expression of MCL-1 and BFL-1 were normalized to β-actin (delta Ct) prior to comparative analysis (delta delta Ct). Graphs were generated and statistical analyses performed using Graphpad Prism 10 (Boston, MA, USA). 2-3 replicates of the same cell line or cell culture were used where indicated; for RT-PCR, 4 technical replicates were averaged for use in fold change calculations. Outliers in RT-PCR technical replicates were omitted if they deviated by more than 2 standard deviations from the average of the other 3 replicates (applies in 1 case). Experiment shown in **Figure 1D-E** was conducted twice, the first being a preliminary experiment run in singlicate, with similar results observed for both iterations. Experiment shown in **Figure S3** was conducted once. Number of replicates for these experiments was determined appropriate based on degree of variation between replicates of the same cell line. Comparisons between two groups were conducted using a paired or unpaired parametric t-test (the former for data in which paired values from one sample were compared) as indicated in figure legends and multiple comparisons were corrected for using the Holm-Šídák method.

**References**

1. Mercier FE, Sykes DB, Scadden DT. Single Targeted Exon Mutation Creates a True Congenic Mouse for Competitive Hematopoietic Stem Cell Transplantation: The C57BL/6-CD45.1STEM Mouse. Stem Cell Rep. 2016;6(6):985–92.

2. Wang GG, Calvo KR, Pasillas MP, Sykes DB, Häcker H, Kamps MP. Quantitative production of macrophages or neutrophils ex vivo using conditional Hoxb8. Nat Methods. 2006;3(4):287–93.

3. Jutz S, Hennig A, Paster W, Asrak Ö, Dijanovic D, Kellner F, et al. A cellular platform for the evaluation of immune checkpoint molecules. Oncotarget. 2017;8(39):64892–906.

4. Xie X, Shi Q, Wu P, Zhang X, Kambara H, Su J, et al. Single-cell transcriptome profiling reveals neutrophil heterogeneity in homeostasis and infection. Nat Immunol. 2020;21(9):1119–33.

5. Fan J, Salathia N, Liu R, Kaeser GE, Yung YC, Herman JL, et al. Characterizing transcriptional heterogeneity through pathway and gene set overdispersion analysis. Nat Methods. 2016;13(3):241–4.

6. Barkas N, Petukhov V, Nikolaeva D, Lozinsky Y, Demharter S, Khodosevich K, et al. Joint analysis of heterogeneous single-cell RNA-seq dataset collections. Nat Methods. 2019;16(8):695–8.

**Supplemental Tables**

**Table S1**: Flow cytometry reagents

| **Antigen** | **Color** | **Clone** | **Working dilution** | **Company** | **Catalog #** |
| --- | --- | --- | --- | --- | --- |
| Viability | DAPI | N/A | 1 ug/mL | Thermo Scientific | 62248 |
| CD11b | APC | M1/70 | 1 to 100 | Biolegend | 101212 |
| GR-1 | BV421 | RB6-8C5 | 1 to 100 | Biolegend | 108445 |
| CXCR2 (CD182) | APC | SA044G4 | 1 to 100 | Biolegend | 149312 |
| CXCR4 (CD184) | APC | L276F12 | 1 to 100 | Biolegend | 146507 |
| CD45.1 | BV421 | A20 | 1 to 400 | Biolegend | 110731 |

**Table S2**: Statistical comparisons for NF-kB pathway expression across clusters, ns (not significant) p>0.05, **p<0.01, ****p<0.0001.

| **.y.** | **group1** | **group2** | **p** | **p.adj** | **p.format** | **p.signif** | **method** |
| --- | --- | --- | --- | --- | --- | --- | --- |
| gene | G0 | G1 | 2.382199e-21 | 1.4e-20 | <2e-16 | **** | Wilcoxon |
| gene | G0 | G2 | 9.231097e-21 | 4.6e-20 | <2e-16 | **** | Wilcoxon |
| gene | G0 | G3 | 9.318665e-23 | 6.5e-22 | <2e-16 | **** | Wilcoxon |
| gene | G0 | G4 | 6.502103e-03 | 2.6e-02 | 0.0065 | ** | Wilcoxon |
| gene | G0 | G5 | 1.415091e-46 | 1.4e-45 | <2e-16 | **** | Wilcoxon |
| gene | G1 | G2 | 5.982170e-01 | 1.0e+00 | 0.5982 | ns | Wilcoxon |
| gene | G1 | G3 | 6.568988e-01 | 1.0e+00 | 0.6569 | ns | Wilcoxon |
| gene | G1 | G4 | 1.826975e-25 | 1.5e-24 | <2e-16 | **** | Wilcoxon |
| gene | G1 | G5 | 3.665973e-83 | 4.8e-82 | <2e-16 | **** | Wilcoxon |
| gene | G2 | G3 | 3.664474e-01 | 1.0e+00 | 0.3664 | ns | Wilcoxon |
| gene | G2 | G4 | 7.267170e-32 | 6.5e-31 | <2e-16 | **** | Wilcoxon |
| gene | G2 | G5 | 2.043125e-119 | 2.9e-118 | <2e-16 | **** | Wilcoxon |
| gene | G3 | G4 | 1.305750e-52 | 1.4e-51 | <2e-16 | **** | Wilcoxon |
| gene | G3 | G5 | 1.351618e-271 | 2.0e-270 | <2e-16 | **** | Wilcoxon |
| gene | G4 | G5 | 1.323033e-72 | 1.6e-71 | <2e-16 | **** | Wilcoxon |

**Supplemental figure legends**

**Figure S1.** Assessing dynamic range of NF-kB-inducible GFP (NG) cell line by stimulating with lipopolysaccharide (LPS).

A) NG cells cultured in expansion medium (+E2) were treated overnight (~16h) with 0.5 or 1 ng/mL LPS and analyzed for GFP expression using flow cytometry. Parental refers to untransduced, GFP^NEG^ ERHOXB8 cells. Shown are live (DAPI^NEG^) singlets. B) Median fluorescence intensity (MFI) values from (A).

**Figure S2.** Increasing constitutive NF-kB activity as cells mature in the absence of E2.

Percent GFP positivity was evaluated at 12h increments as cells mature in the absence of β-estradiol using flow cytometry. Shown are singlets.

**Figure S3.** Ability of murine ERHOXB8 neutrophils to phagocytose *C. albicans* does not appear to depend on NF-kB activation.

A) Flow cytometry was used to assess phagocytosis of iRFP-labeled *C. albicans* in relation to GFP positivity at 12h increments as NG cells mature in the absence of β-estradiol. Shown are CD45.1^POS^ singlets. Cells were challenged with *C. albicans* at a multiplicity of infection (MOI) of 4 *C. albicans* per neutrophil. B) Quantification from (A), given as the ratio of cells phagocytosing to cells not phagocytosing for GFP^NEG^ (upper left quadrant / lower left quadrant) and GFP^POS^ (upper right quadrant / lower right quadrant). C) Total proportion of GFP^POS^ cells in *C. albicans* challenged (from (A), upper right quadrant + lower right quadrant) and corresponding resting or unchallenged samples. N = 3 independently challenged wells from the same cell culture for phagocytosis assay; N = 2 for resting samples from (C). Comparisons made using a paired, parametric t-test (p=0.43, 0.43, 0.43, 0.36, 0.36, 0.28, 0.43, 0.23, 0.17, 0.31 for 0, 12, 24, 36, 48, 60, 72, 84, 96, and 108h, respectively) (B) or unpaired, parametric t-test (p=0.85, 0.61, 0.43, 0.85, 0.43, 0.85 0.17, 0.11, 0.36, 0.59 for 0, 12, 24, 36, 48, 60, 72, 84, 96, and 108h, respectively) (C). Multiple comparisons were corrected for using the Holm-Šídák method, ns (not significant) p>0.05. Shown is mean ± SD.

**Figure S4.** Surface expression of CXCR2 and CXCR4 does not necessarily follow the onset of NF-kB activation.

A) Surface expression of CD182 / CXCR2 in relation to GFP positivity was evaluated at 12h increments as cells mature in the absence of β-estradiol using flow cytometry. Shown are singlets. B) As in (A), but for CD184 / CXCR4.
